# A novel bit-quad-based Euler number computing algorithm

**DOI:** 10.1186/s40064-015-1511-8

**Published:** 2015-11-25

**Authors:** Bin Yao, Lifeng He, Shiying Kang, Yuyan Chao, Xiao Zhao

**Affiliations:** Artificial Intelligence Institute, College of Electrical and Information Engineering, Shaanxi University of Science and Technology, Xi’an, 710021 Shaanxi China; Faculty of Information Science and Technology, Aichi Prefectural University, Aichi, 4801198 Japan; School of Information Engineering, Xianyang Normal University, Xianyang, 712000 Shaanxi China; Faculty of Environment, Information and Business, Nagoya Sangyo University, Aichi, 4888711 Japan

**Keywords:** Euler number, Graph theory, Computer vision, Pattern recognition, Topological property

## Abstract

The Euler number of a binary image is an important topological property in computer vision and pattern recognition. This paper proposes a novel bit-quad-based Euler number computing algorithm. Based on graph theory and analysis on bit-quad patterns, our algorithm only needs to count two bit-quad patterns. Moreover, by use of the information obtained during processing the previous bit-quad, the average number of pixels to be checked for processing a bit-quad is only 1.75. Experimental results demonstrated that our method outperforms significantly conventional Euler number computing algorithms.

## Background

The topological properties of binary images are very useful features in the fields of pattern recognition and computer vision. Among others, the Euler number of a binary image, which is defined as the difference between the number of connected components and that of holes in the image, is one of the most important topological properties (Gonzalez and Woods 2008). The Euler number of a binary image will not change when the image is stretched, flexed or rotated. Therefore, the Euler number has been used in many applications: processing cell images in medical diagnosis (Hashizume et al. 1990), document image processing (Srihari 1986), shadow detection (Rosin and Ellis 1995), reflectance-based object recognition (Nayar and Bolle 1996), and robot vision (Horn 1986). Moreover, the Euler number is the most clinically useful feature for discriminating many cervical disorders (Pogue et al. 2000).

In the past decades of years, many algorithms have been proposed for computing the Euler number of a binary image. For example, there are skeleton-based algorithm (Diaz-de-Leon and Sossa-Azuela 1996), which calculates the Euler number by use of the number of terminal points and the number of three edge points in the corresponding skeleton image; bit-quad-based algorithm proposed by Gray (1971), which calculates the Euler number by counting certain 2 × 2 pixel patterns called bit-quads, and is adopted by the famous commercial image processing tools MATLAB (Thompson and Shure 1995). There are also run-based algorithm (Bishnu et al. 2005), which calculates the Euler number by use of the numbers of runs and the neighboring runs in the image, and labeling-based algorithm proposed by He et al. (2013), which calculates the Euler number by labeling connected components and holes in the image. Recently, an improved bit-quad-based algorithm was proposed (Yao et al. 2014), which reduces the number of pixels to be checked for processing a bit-quad from 4 to 2. For convenience, we denote the algorithms proposed in Ref. (Gray 1971), Ref. (Bishnu et al. 2005), Ref. (He et al. 2013) and Ref. (Yao et al. 2014) as *GRAY algorithm, RUN algorithm, HCS algorithm and I*-*GRAY algorithm*, respectively.

On the other hand, there are also parallel algorithm (Chiavetta and Gesu 1993), hardware algorithm (Dey S. et al. 2000), and algorithms for images with quad-tree represented formats (Dyer 1980), (Samet and Tamminen 1985). In recent years, other algorithms have been proposed for computing the Euler number in a binary image. For example, Sossa-Azuelal proposed the algorithm for computing Euler number based on a vertex codification (Sossa-Azuelal et al. 2013), and he also proposed an alternative algorithm in (Sossa-Azuela et al. 2014). Yao (2015) improve the Euler number computing algorithm based on runs and neighboring runs. He and Chao (2015) proposed an algorithm for labeling connected-component and computing Euler number simultaneously.

This paper presents a novel bit-quad-based Euler number computing algorithm. Based on graph theory, instead of counting ten bit-quad patterns in conventional bit-quad-based algorithms, our algorithm only needs to count two bit-quad patterns for Euler number computing. Moreover, by use of the information obtained during processing the previous bit-quad similar as in the I-GRAY algorithm, the average number of pixels to be checked for processing a bit-quad is reduced to 1.75, which leads to more efficient processing. Experimental results showed that our algorithm is much more efficient than conventional Euler number computing algorithms on various kinds of images.

The rest of this paper is organized as follows. In “Reviews of related conventional Euler number computing algorithms”, we review related conventional Euler number computing algorithms. We propose our algorithm in “Our proposed algorithm”, present experimental results in “Experimental results”, and make a discussion in “Discussion”. Lastly, we give our conclusion in “Conclusion”.

## Reviews of related conventional Euler number computing algorithms

For an *M* × *N*-size binary image, we use *p*(*x*, *y*) to denote the pixel at (*x*, *y*), where 1 ≤ *x* ≤ *M*, 1 ≤ *y* ≤ *N*. As in most image processing algorithms, we assume that the object (foreground) pixels and background pixels in a given binary image are represented by 1 and 0 respectively, and all pixels on the border of an image are background pixels. Moreover, we only consider 8-connectivity in this paper.

### GRAY algorithm

**Fig. 1:**
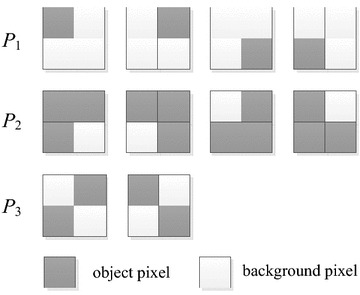
Patterns of bit-quads for calculating the Euler number in the GRAY algorithm

The GRAY algorithm (Gray 1971) for calculating the Euler number of a binary image is based on counting certain 2 × 2 pixel patterns called bit-quads. While computing the Euler number of a binary image, it needs to scan the image from left to right and from top to bottom. In the scanning, each pixel and other three pixels in the corresponding bit-quad need to be checked for finding the patterns of the bit-quad shown in Fig. 1. For example, for the pixel *p*(*x*, *y*) in the image, it checks whether the corresponding bit-quad, i.e., $$\left[ {\begin{array}{*{20}c} {\rm{{p}(x} - 1, \rm{y} - 1)} & {\rm{p(x, y} - 1)} \\ {\rm{{p}(x} - 1, \rm{y})} & {\text{p(x, y)}} \\ \end{array} } \right]$$, is one of patterns *P*_1_, *P*_2_, and *P*_3_. When the scanning is completed, we can obtain the numbers of patterns *P*_1_, *P*_2_, and *P*_3_. Let *N*_1_, *N*_2_, and *N*_3_ be the numbers of patterns *P*_1_, *P*_2_, and *P*_3_ in the image, respectively, then, the Euler number of the image, denoted as *E*, can be calculated by the following formula.

1 $$E = \left( {N_{ 1} -N_{ 2} - 2N_{ 3} } \right)/ 4$$

Obviously, for processing a pixel, it will take four pixel accesses in a bit-quad in the GRAY algorithm. Thus, for calculating the Euler number of an *M* × *N*-size binary image, it will take 4 × *M* × *N* pixel accesses in total.

### RUN algorithm

The RUN algorithm (Bishnu et al. 2005) calculates the Euler number by use of the number of runs and the number of neighboring runs in the given image.

**Fig. 2:**
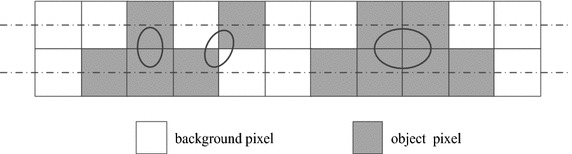
An example for explaining the runs and the neighboring runs

A run is defined to be a maximal sequence of consecutive object pixels in a row. A run *R*_1_ is said to be a neighboring run of another run *R*_2_ if there is at least a pixel in *R*_1_ such that it is 8-connected with a pixel in *R*_2_. For example, in Fig. 2, there are three runs in the first row, two runs in the second row and three neighboring runs marked by black oval shape between two rows. We denote the numbers of runs and neighboring runs as *R* and *O* in the given image, respectively.

Having counted all runs and neighboring runs in the given image, the Euler number of the image can be calculated by the following formula.

2 $$E = R-O$$

### HCS algorithm

The HCS algorithm (He et al. 2013) calculates the Euler number of a binary image according to the definition of the Euler number:

3 $$E \, = \, C \, - \, H$$

where *C* is the number of the connected components, and *H* is that of the holes in the image, respectively.

For calculating *C* and *H*, this algorithm extended the labeling algorithm proposed in Ref. (He et al. 2010) to label connected components and holes in the binary image simultaneously. At any moment in the raster scan, all provisional labels assigned to an 8-connected component or a 4-connected hole in the processed area of the image are combined in an equivalent label set, respectively. Thus, after the raster scan, all provisional labels assigned to a connected component or a hole in the image will be combined in an equivalent label set, respectively. Then, by counting the number of the equivalent label sets corresponding to connected components and that for holes, we can obtain the number of connected components, i.e., *C*, and that of holes, i.e., *H*, respectively.

### I-GRAY algorithm

The I-GRAY algorithm (Yao et al. 2014) is an improvement on the GRAY algorithm. It also needs to process all bit-quads in the given image and count the number of the special bit-quad patterns in the same way as in the GRAY algorithm. However, by use of the already-known information obtained during processing the previous pixel, it reduces the number of pixels necessary to be checked for processing a bit-quad from 4 to 2.

## Our proposed algorithm

As one of topological properties, the Euler number of a binary image can also be calculated according to graph theory. Chen and Yan proposed a graph-based algorithm for calculating the Euler number of a binary image for 4-connectivity (Chen and Yan 1988) by counting all vertices, edges and faces in the graph corresponding to the image. In this section, we first introduce how to use graph theory to calculate the Euler number of a binary image for 8-connectivity. Then, we show that only two kinds of bit-quad patterns need to be considered for calculating the Euler number.

**Fig. 3:**
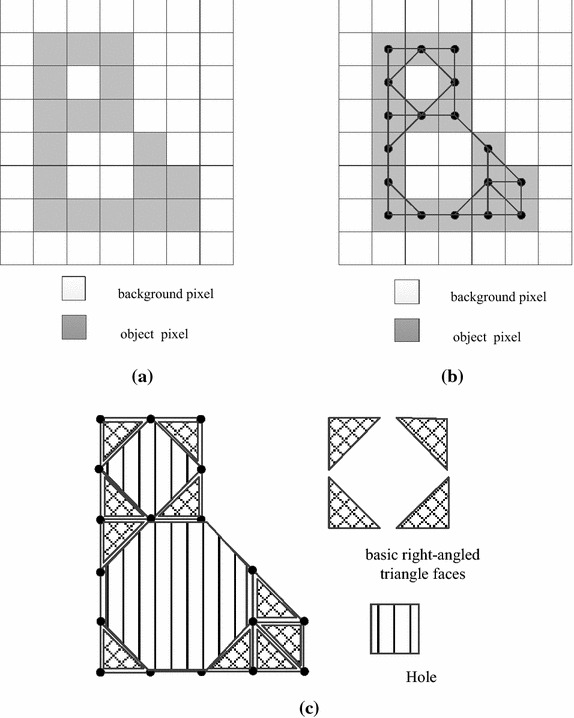
An example for constructing a graph corresponding to a binary image

In order to use graph theory to calculate the Euler number of a binary image, we construct a square graph corresponding to the image. To do that, we take all object pixels in the image as vertices and add an edge between two object pixels if and only if they are 8-connected neighbors for each other unless the edge crosses with another edge. For example, for the given image shown in Fig. 3a, according to the constructing method, the vertices and edges can be added as in Fig. 3b. Thus, we can obtain a square graph corresponding to the image as shown in Fig. 3c.

Euler’s theorem in graph theory can be described as follows (West 2001).

### Euler’s theorem

If *G* is a square graph, *v*, *e*, *r* and *C* are the numbers of vertices, edges, squares and the connected components in *G*, respectively. Then, *v* − *e* + *r* = *C* + 1.

In Euler’s theorem, the squares in graph *G* include holes, basic faces and an infinite square outside of *G*. Let *H* and *s* be the number of holes and basic faces in graph *G*, respectively. Accordingly, *r* = *H* + *s* + 1. Then we have *v* − *e* + (*H* + *s* + 1) = *C* + 1. Thus, the Euler number *E* can be represented as:

4 $$E \, = \, C-H = v-e + s$$

In this way, we can calculate the Euler number of a binary image by use of the numbers of vertices, edges and basic faces in its corresponding graph. Notice that in the case of 8-connectivity, the number of basic faces *s* in the formula (4) refers to the number of basic right-angled triangle faces.

In practice, when using the formula (4) to calculate the Euler number of a binary image, we can count the number of vertices, edges, and basic faces without constructing a corresponding square graph but by checking all bit-quads in the given image.

**Fig. 4:**
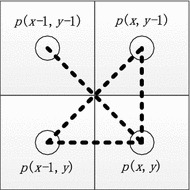
A diagram for processing a bit-quad

Obviously, the number of vertices in the corresponding graph is equal to the number of object pixels in the image. For a bit-quad shown in Fig. 4, pixel *p*(*x*, *y*) is said to be *the representative pixel* of the bit-quad. For convenience, a bit-quad with *q* as the representative pixel is denoted as *B*(*q*). It is obvious that only if pixel *p*(*x*, *y*) is an object pixel, the number of vertices will be increased by 1. Notice that the vertex corresponding to each of other object pixels in the bit-quad, says, *t*, has been considered when processing the bit-quad *B*(*t*).^1^

On the other hand, for calculating the number of new edges in the bit-quad, we should consider whether there are edges between *p*(*x*, *y*) and *p*(*x*, *y* − 1), *p*(*x*, *y*) and *p*(*x* − 1, *y*), *p*(*x*, *y*) and *p*(*x* − 1, *y* − 1), and *p*(*x* − 1, *y*) and *p*(*x*, *y* − 1), respectively. Notice that whether there are edges between *p*(*x* − 1, *y*) and *p*(*x* − 1, *y* − 1), and *p*(*x* − 1, *y* − 1) and *p*(*x*, *y* − 1) have already been considered when processing *B*(*p*(*x* − 1, *y*)) and *B*(*p*(*x*, *y* − 1)), respectively. Furthermore, in the case where both edges *p*(*x*, *y*) and *p*(*x* − 1, *y* − 1), and *p*(*x* − 1, *y*) and *p*(*x*, *y* − 1) might exist, only one should be considered. Because there is an edge between *p*_1_ and *p*_2_ if and only if *p*_1_ and *p*_2_ are object pixels, the rules for calculating the number of edges can be shown as follows, where *e*(*u*, *v*) denotes the edge between object pixels *u* and *v*.

**Fig. 5:**
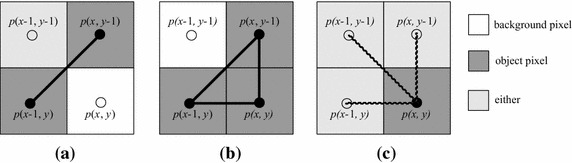
Illustration for calculating the number of edges in a bit-quad
- If *p*(*x*, *y*) is an object pixel, in the case where *p*(*x* − 1, *y*) and *p*(*x*, *y* − 1) are object pixels and *p*(*x* − 1, *y* − 1) is a background pixel (Fig. 5b), the three edges *e*(*p*(*x*, *y*), *p*(*x* − 1, *y*)), *e*(*p*(*x*, *y*), *p*(*x*, *y* − 1)), and *e*(*p*(*x* − 1, *y*), *p*(*x*, *y* − 1)) should be counted; in the other cases, for each object pixel *q* among pixels *p*(*x* − 1, *y*), *p*(*x*, *y* − 1) and *p*(*x* − 1, *y* − 1), an edge *e*(*p*(*x*, *y*), *q*) should be counted (Fig. 5c).

**Fig. 6:**
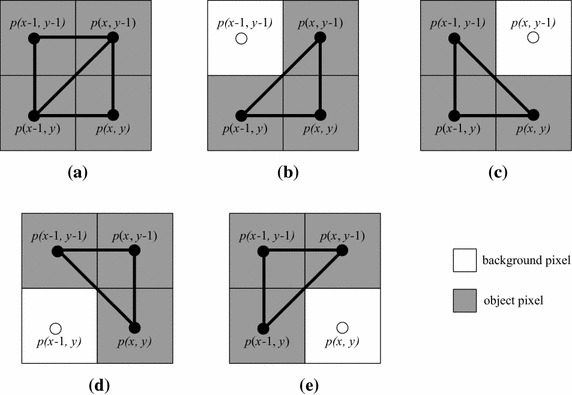
Illustration for calculating the number of basic *right-angled triangle* faces in a bit-quad

- If *p*(*x*, *y*) is a background pixel, no edge between *p*(*x*, *y*) and *p*(*x*, *y* − 1), between *p*(*x*, *y*) and *p*(*x* − 1, *y*), and between *p*(*x*, *y*) and *p*(*x* − 1, *y* − 1). On the other hand, when and only when both *p*(*x* − 1, *y*) and *p*(*x*, *y* − 1) are object pixels, the edge *e*(*p*(*x* − 1, *y*), *p*(*x*, *y* − 1)) should be counted (Fig. 5a);

As for calculating the number of basic right-angled triangle faces in the bit-quad, we only need to check the number of object pixels in the bit-quad. The number of basic right-angled triangle faces will be two if all pixels in the bit-quad are object pixels (Fig. 6a), and one if there are three object pixels (Fig. 6b–e). Otherwise, no basic right-angled triangle face exists.

When all bit-quads in the given image are processed, we can obtain the number of vertices, edges and basic right-angled triangle faces in the corresponding graph, and calculate the Euler number of the image by use of formula (4) easily.

However, calculating the Euler number of a binary image by counting the numbers of vertices, edges and faces directly will be inefficient. In order to do this work more efficiently, we analyze all 16 patterns of a bit-quad. For each pattern, according to the above calculating methods, we can obtain the increments of the numbers of vertices, edges and faces, and the Euler number, which are denoted by ∆*v*, ∆*e*, ∆*s*, and ∆*E*, respectively, where ∆*E* = ∆*v* − ∆*e* + ∆*s*, as shown in Table 1.

**Table 1:**
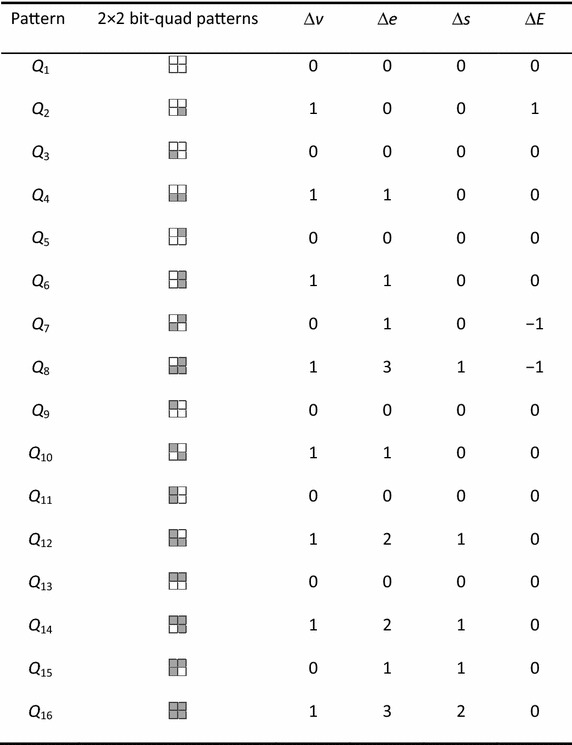
The increments of the numbers of *v*, *e* and *s*, and the Euler number Δ*E* for a bit-quad pattern

**Fig. 7:**
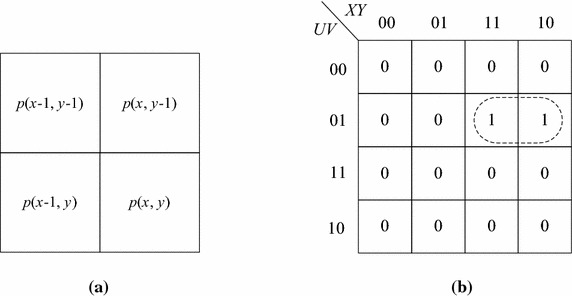
A bit-quad to be processed and the Karnaugh map for a bit-quad to be pattern *Q*_7_ or *Q*_8_

**Fig. 8:**
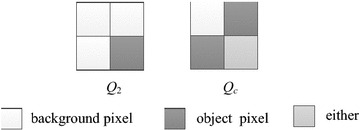
Bit-quad patterns used in our algorithm

According to Table 1, when processing a bit-quad shown in Fig. 7a, the Euler number will increase by 1 only when it is pattern *Q*_2_, and will decrease by 1 only if it is either pattern *Q*_7_ or pattern *Q*_8_. Obviously, the conditions for a bit-quad to be pattern *Q*_2_ are that the representative pixel is object pixel and all other pixels in the bit-quad are background pixels. On the other hand, the conditions for a bit-quad to be patterns *Q*_7_ or *Q*_8_, which can be derived by use of the Karnaugh map (Karnaugh 1953) shown in Fig. 7b, are that *p*(*x* − 1, *y* − 1) is a background pixel, and *p*(*x*, *y* − 1) and *p*(*x* − 1, *y*) are object pixels. Notice that it does not matter whether the representative pixel *p*(*x*, *y*) is an object pixel or not. Therefore, we can combine the two patterns *Q*_7_ and *Q*_8_ to one pattern *Q*_*c*_, as shown in Fig. 8. Thus, let *W*_2_ and *W*_*c*_ be the numbers of *Q*_2_ and *Q*_*c*_ in the given image, respectively, we can use the following formula to calculate the Euler number of the image.

5 $$E \, = \, W_{ 2} - W_{c}$$

Now we introduce how to check whether a bit-quad is a pattern of *Q*_2_ or *Q*_*c*_ when processing the given image in the raster scan.

If *p*(*x* − 1, *y* − 1) is an object pixel, the current bit-quad will be neither *Q*_2_ nor *Q*_*c*_, so we can skip the bit-quad and go to process the next bit-quad.

**Fig. 9:**
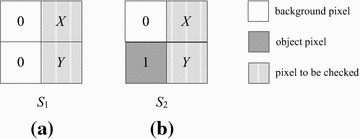
Two states for the case where pixel *p*(*x*-1, *y*-1) is 0 in our algorithm

If *p*(*x* − 1, *y* − 1) is a background pixel, we need to check other pixels in the bit-quad. Because *p*(*x* − 1, *y*) is either 0 or 1, there are two states as shown in Fig. 9.

For state *S*_1_, we need to check both pixel *X* and pixel *Y*. There are following three cases: (1) if pixel *X* is 1, the current bit-quad and the next bit-quad to be processed will be none of patterns *Q*_2_ and *Q*_*c*_, we do nothing else for the current bit-quad and skip the next bit-quad over; (2) if both pixel *X* and pixel *Y* are 0, the current bit-quad will be none of patterns *Q*_2_ and *Q*_*c*_, then we go to process the next bit-quad, which obviously will be a case of state *S*_1_ (Fig. 9a); (3) if pixel *X* is 0 and pixel *Y* is 1, the current bit-quad is pattern *Q*_2_, thus, *W*_2_ increases by 1, then we go to process the next bit-quad, which will be a case of state *S*_2_ (Fig. 9b).

For state *S*_2_, we also need to check pixel *X* and pixel *Y*. There are the following three cases: (1) if pixel *X* is 1, the current bit-quad is pattern *Q*_*c*_, thus, *W*_*c*_ increases by 1. At the same time, we know the next bit-quad will be none of patterns *Q*_2_ or *Q*_*c*_, so we can skip the next bit-quad over; (2) if both pixel *X* and pixel *Y* are 0, the current bit-quad will be none of patterns *Q*_2_ and *Q*_*c*_, then we go to process the next bit-quad, which will be a case of state *S*_1_; (3) if pixel *X* is 0 and pixel *Y* is 1, the current bit-quad will be none of patterns *Q*_2_ or *Q*_*c*_, then we go to process the next bit-quad, which will be a case of state *S*_2_.

After processing all bit-quads in the given image, we can obtain the numbers of the patterns *Q*_2_ and *Q*_*c*_, i.e., *W*_2_ and *W*_*c*_, then, we can calculate the Euler number by use of the formula (5).

The pseudo codes of our algorithm can be shown as follows. 

## Experimental results

Images used for evaluating the algorithms were composed of artificial images (including 41 noise images and 4 specialized pattern images), 50 natural images obtained from the Standard Image Database (SIDBA) developed by the University of Tokyo^2^ and the image database of the University of Southern California,^3^ 7 texture images downloaded from the Columbia-Utrecht Reflectance and Texture Database,^4^ and 25 medical images obtained from a medical image database of the University of Chicago.

In the experiments, we compared our algorithm with the GRAY algorithm, the RUN algorithm, the HCS algorithm, and the I-GRAY algorithm. All algorithms used for our comparison were implemented in the C language on a PC-based workstation (Intel Core i5-3470 CPU@3.20 GHz, 4 GB Memory, Ubuntu Linux OS), and compiled by the GNU C compiler (version 4.2.3) with the option –O. All experimental results presented in this section were obtained by averaging of the execution time for 5000 runs.

### Execution time versus the density of an image

**Fig. 10:**
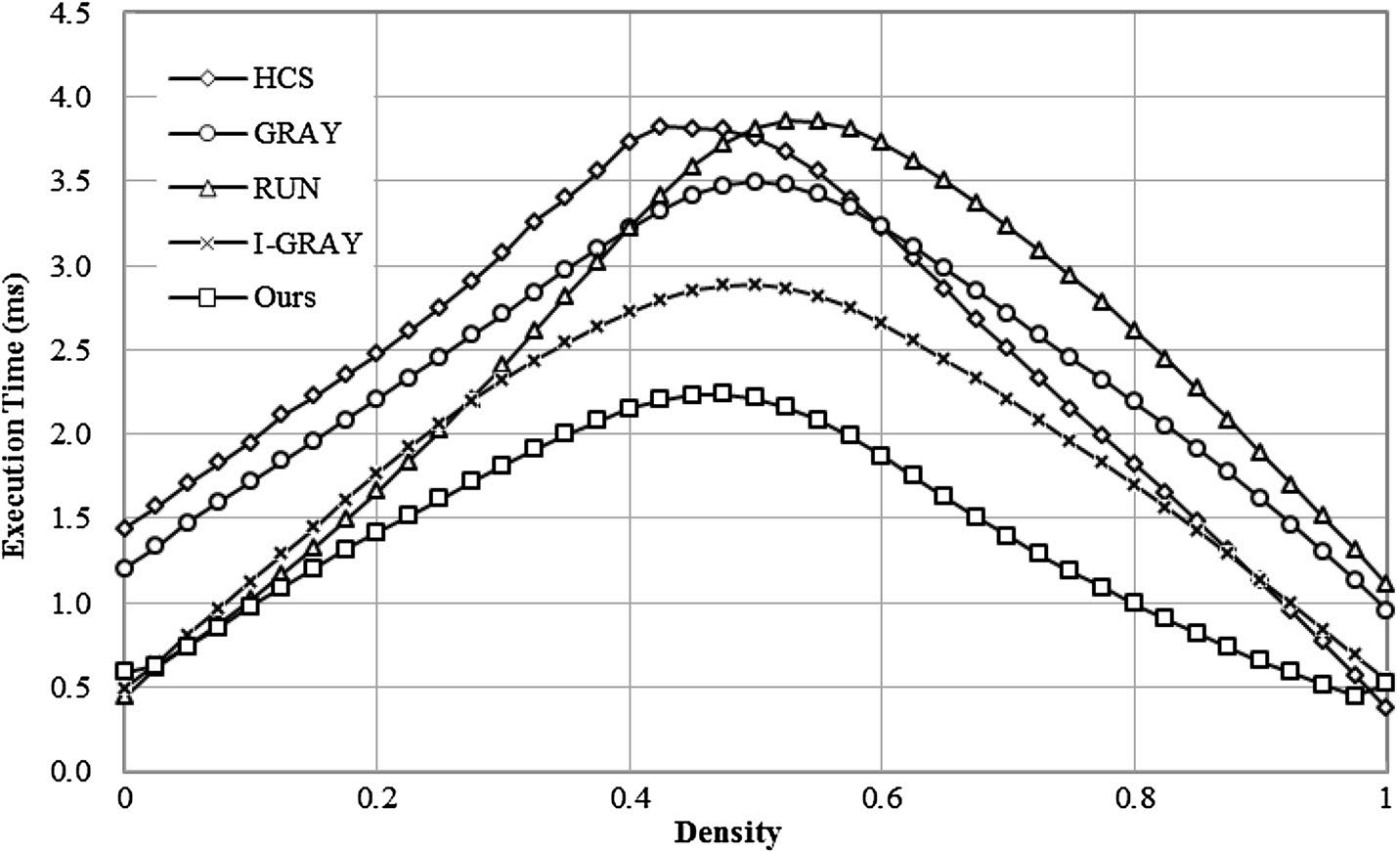
Execution time versus density of an image

Because connected components in noise images have complicated geometric shapes and complex connectivity, severe evaluations of algorithms can be performed with these images. 41 noise images with a size of 512 × 512 pixels, which were generated by thresholding of the images containing uniform random noise with 41 different threshold values from 0 to 1000 in steps of 25, were used for testing the execution time versus the density of the foreground pixels^5^ in an image. The results are shown in Fig. 10. We can find that our algorithm is much better than the GRAY algorithm for all images, is better than the HCS algorithm for all images except for the images whose densities are over 97 %, and is also much better than the RUN algorithm and the I-GRAY algorithm for all images whose densities are over 5 %.

### Comparisons in terms of the maximum, mean, and minimum execution times on various kinds of real images

In this test, all the 50 natural images, 25 medical images, 7 texture images, and 4 artificial images with specialized shape patterns (saw-tooth-like, checker-board-like, stair-like, and honey comb-like connected components) were used for evaluating the algorithms. The resolution of all of these images is 512 × 512 pixels. The results are shown in Table 2.

**Table 2:** Maximum, mean, and minimum execution times (ms) on various types of images

Image Type	GRAY	RUN	HCS	I-GRAY	Ours
Natural					
Max.	1.86	1.69	1.97	1.34	1.02
Mean	1.42	1.07	1.40	0.86	0.71
Min.	1.10	0.61	0.87	0.55	0.49
Medical					
Max.	1.47	1.07	1.50	0.89	0.73
Mean	1.29	0.92	1.25	0.72	0.62
Min.	1.17	0.75	0.91	0.63	0.54
Textural					
Max.	1.73	1.66	1.60	1.16	0.92
Mean	1.38	1.35	1.10	0.83	0.68
Min.	1.00	1.04	0.51	0.49	0.51
Artificial					
Max.	1.11	1.03	1.35	0.56	0.49
Mean	0.70	0.67	0.70	0.35	0.28
Min.	0.28	0.24	0.32	0.16	0.11

**Fig. 11:**
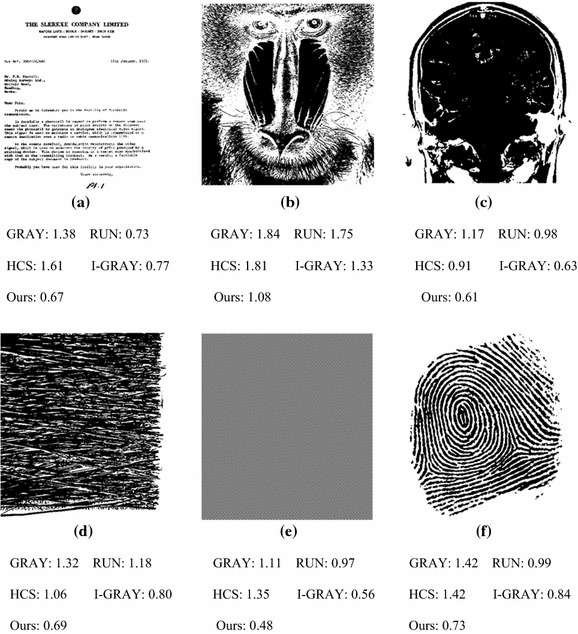
Execution time (*ms*) for the selected six images: **a** a text image; **b** a portrait image; **c** a medical image; **d** a texture image; **e** an artificial image; **f** a fingerprint image

From Table 2, for all types of images, our algorithm is much more efficient than both of the GRAY algorithm and the RUN algorithm for all of the minimum time, the average time and the maximum time. Compared to the I-GRAY algorithm and the HCS algorithm, our algorithm is more efficient than either of the two algorithms for the average time and the maximum time. In fact, for the images used in this test, our algorithm is better than any of the other algorithms in comparison except for one texture image. The execution time (ms) for the selected six images are illustrated in Fig. 11, where the object pixels are displayed in black.

## Discussion

### Other groups of patterns for calculating the Euler number

**Fig. 12:**
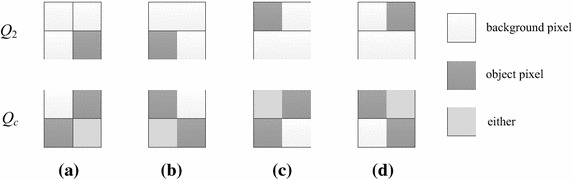
Four groups of patterns used for calculating the Euler number

According to the analysis in “Our proposed algorithm”, we can calculate the Euler number of a binary image by the numbers of the bit-quad patterns in the image shown in Fig. 12a. Because the Euler number of a binary image will not change when the image is rotated, therefore, for a binary image, if we rotate it by 90°, 180° and 270° clockwise, the bit-quad patterns *Q*_2_ and *Q*_*c*_ needed to be counted will become to the patterns shown in Fig. 12b–d, respectively. Theoretically, we can use any of the groups of the patterns to compute the Euler number of a binary image.

### Time complexity

According to the analysis results given in related references, for calculating the Euler number of an *M* × *N*-size binary image, the skeleton-based algorithm will take about 8 *M* × *N* pixel accesses (Diaz-de-Leon and Sossa-Azuela 1996), the GRAY algorithm will take 4 *M* × *N* pixel accesses, the RUN algorithm will take about 4 *M* × *N* pixel accesses in the worst case, and about 3 *M* × *N* pixel accesses in average (Bishnu et al. 2005). Moreover, the HCS algorithm will take 2.375 *M* × *N* pixel accesses in average (He et al. 2013). Taking advantage of the information obtained during processing the previous bit-quad, the I-GRAY algorithm will only take 2 *M* × *N* pixel accesses (Yao et al. 2014). Therefore, the I-GRAY algorithm is better than the skeleton-based algorithm, the GRAY algorithm, the RUN algorithm, and the HCS algorithm.

In our algorithm, as introduced in “Our proposed algorithm”, for processing a bit-quad $$\left[ {\begin{array}{*{20}c} U & X \\ V & Y \\ \end{array} } \right]$$, the pixels in the bit-quad will be checked in the order *U* → *V* → *X* → *Y*. If *U* is an object pixel, i.e., the bit-quad is $$\left[ {\begin{array}{*{20}c} 1 & X \\ V & Y \\ \end{array} } \right]$$ (the patterns *Q*_9_–*Q*_16_ in Table 3), denoted as *R*1, we will do nothing else. Thus, we only need to check one pixel. Otherwise, if *U* is a background pixel, we will check *V* and *X*. For a bit-quad such as $$\left[ {\begin{array}{*{20}c} 0 & 1 \\ 0 & Y \\ \end{array} } \right]$$ or $$\left[ {\begin{array}{*{20}c} 0 & 1 \\ 1 & Y \\ \end{array} } \right]$$ (the patterns *Q*_5_–*Q*_8_ in Table 3), denoted as *R*2, we need to check three pixels, but we can skip over the next bit-quad, thus, we need to check 1.5 pixels for processing a bit-quad in average. For each of the rest patterns such as $$\left[ {\begin{array}{*{20}c} 0 & 0 \\ 1 & 0 \\ \end{array} } \right]$$, $$\left[ {\begin{array}{*{20}c} 0 & 0 \\ 1 & 1 \\ \end{array} } \right]$$, $$\left[ {\begin{array}{*{20}c} 0 & 0 \\ 0 & 1 \\ \end{array} } \right]$$ or $$\left[ {\begin{array}{*{20}c} 0 & 0 \\ 0 & 0 \\ \end{array} } \right]$$ (the patterns *Q*_1_–*Q*_4_ in Table 3), denoted as *R*3, we need to check two pixels for processing the bit-quad if it follows another such a pattern of *R*3. Otherwise, all the four pixels in the bit-quad will be checked. Suppose that all patterns of bit-quads occur in same probability, then, the probability that a pattern of *R*3 follows another pattern of *R*3 is 4/16 = 1/4. Thus, the average number of pixels to be checked for processing a bit-quad of pattern *R*3 will be 2 × 4/16 + 4 × 12/16 = 3.5.

**Table 3:**
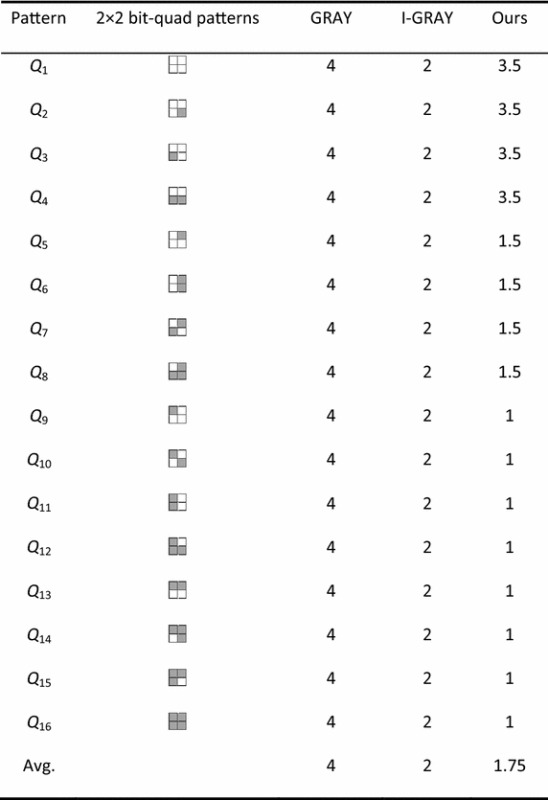
The number of pixels needs to be checked in every bit-quad pattern in the GRAY algorithm, the I-GRAY algorithm and our algorithm

According to above analysis, by our algorithm,the average number of pixels to be checked for processing a bit-quad will be (1 × 8 + 1.5 × 4 + 3.5 × 4)/16 = 1.75. Thus, for an *M* × *N*-size binary image, our algorithm will take about 1.75 *M* × *N* pixel accesses, which is less than the number of pixel accesses in any of conventional Euler number computing algorithms. Therefore, our algorithm will be more efficient than any of conventional algorithms.

The above analysis results are consistent with our experimental results. As mentioned in “Experimental results”, except one image, our algorithm is more efficient than all conventional Euler number computing algorithm in comparison for all images used in our test.

## Conclusion

In this paper, we presented a novel bit-quad-based algorithm for Euler number computing. According to graph theory and analysis on bit-quad patterns, we only need to count two bit-quad patterns, much less than ten patterns counted in conventional bit-quad-based algorithms. Together with use of the information obtained during processing the previous bit-quad, our algorithm checks only 1.75 pixels for processing a bit-quad in average. Experimental results on various types of images demonstrated that our algorithm outperformed conventional Euler number computing algorithms. For future work, we will consider hardware implementation and parallel implementation of our algorithm.

## Footnotes

1 Because pixels in the image are processed in the raster scan, all pixels in the bit-quad except the representative pixels have been processed before processing the representative pixel.

2 The images can be downloaded at http://sampl.ece.ohio-state.edu/data/stills/sidba/index.htm.

3 The images can be downloaded at http://sipi.usc.edu/database/.

4 The images can be downloaded at http://www1.cs.columbia.edu/CAVE/software/curet/.

5 The density of foreground pixels in a binary image refers to the proportion of foreground pixels in the image. Thus, if all pixels in a binary image are background pixels, the density of the image will be 0. On the other hand, if all the pixels are foreground pixels, the density will be 1.
